# Rasch Modeling and Differential Item Functioning of the Self-Stigma Scale-Short Version among People with Three Different Psychiatric Disorders

**DOI:** 10.3390/ijerph19148843

**Published:** 2022-07-21

**Authors:** Chia-Wei Fan, Kun-Chia Chang, Kuan-Ying Lee, Wen-Chi Yang, Amir H. Pakpour, Marc N. Potenza, Chung-Ying Lin

**Affiliations:** 1 Department of Occupational Therapy, AdventHealth University, Orlando, FL 32803, USA; chia-wei.fan@ahu.edu; 2 Department of General Psychiatry, Jianan Psychiatric Center, Ministry of Health and Welfare, Tainan 71742, Taiwan; kunchiachang0517@gmail.com; 3 Department of Child and Adolescent Psychiatry, Jianan Psychiatric Center, Ministry of Health and Welfare, Tainan 71742, Taiwan; ky67122@hotmail.com; 4 Division of Hematology and Medical Oncology, Department of Internal Medicine, E-DA Hospital, Kaohsiung 82445, Taiwan; 5 Faculty of School of Medicine, College of Medicine, I-Shou University, Kaohsiung 84001, Taiwan; 6 Department of Nursing, School of Health and Welfare, Jönköping University, SE-551 11 Jönköping, Sweden; amir.pakpour@ju.se; 7 Department of Psychiatry, Yale School of Medicine, New Haven, CT 06510, USA; marc.potenza@yale.edu; 8 Connecticut Mental Health Center, New Haven, CT 06519, USA; 9 Connecticut Council on Problem Gambling, Wethersfield, CT 06109, USA; 10 Child Study Center, Yale School of Medicine, New Haven, CT 06510, USA; 11 Department of Neuroscience, Yale University, New Haven, CT 06511, USA; 12 Wu Tsai Institute, Yale University, New Haven, CT 06510, USA; 13 Institute of Allied Health Sciences, College of Medicine, National Cheng Kung University, Tainan 70101, Taiwan; 14 Biostatistics Consulting Center, National Cheng Kung University Hospital, College of Medicine, National Cheng Kung University, Tainan 70403, Taiwan; 15 Department of Public Health, College of Medicine, National Cheng Kung University, Tainan 70101, Taiwan; 16 Department of Occupational Therapy, College of Medicine, National Cheng Kung University, Tainan 70101, Taiwan

**Keywords:** self-stigma, substance-related disorders, addictive behaviors, impulsivity, psychotic disorders, Rasch, psychometric testing, validity, differential item functioning

## Abstract

Self-stigma is prevalent in individuals with psychiatric disorders and can profoundly affect people. A unified assessment with sound psychometric properties is needed for evaluating self-stigma across psychiatric conditions. The aim of this study was to examine the psychometric properties of the Self-Stigma Scale-Short version (SSS-S) using Rasch modeling. Six-hundred and twelve participants with substance use disorders (*n* = 319), attention-deficit/hyperactivity disorder (*n* = 100), and schizophrenia (*n* = 193) completed the SSS-S. Rasch results confirmed the unidimensionality of the nine items of the SSS-S. The four-point Likert scale of the SSS-S reflected monotonical increases along the self-stigma continuum. No ceiling or floor effects were detected. Among the three subdomains of the SSS-S, cognitive items appeared to be the most robustly endorsed, and behavioral items were the least endorsed. Two items in the SSS-S displayed differential item functioning across the three diagnoses. Additionally, SSS-S scores showed weak to moderate correlation with depression, anxiety, and stress scale scores. The SSS-S had overall satisfactory psychometric properties. Healthcare professionals may use this assessment to assess self-stigma in multiple psychiatric groups, and information gained may facilitate improved care.

## 1. Introduction

Self-stigma involves the internalization of discriminatory, prejudiced, stereotypical or other negative beliefs regarding one’s personal characteristics [1], which is particularly relevant to minority and disadvantaged groups [1]. Self-stigma is prevalent in psychiatric populations. A prior study found that self-stigma was moderated by knowledge of psychiatric conditions and cultural relevance [2]. A recent systematic review showed that, on average, 31.3% of individuals with psychiatric disorders reported high self-stigma. The highest frequency of self-stigma globally (39.7%) was in Southeast Asia [3]. Among people with mental illness in Hong Kong and Guangzhou, 38.3% to 49.5% reported high levels of self-stigma [4]. Another study showed the overall reported self-stigma prevalence was 54% in Nepal [4].

People with self-stigma have low self-esteem and self-efficacy [5,6]. Livingston and Boyd confirmed a robust relationship between self-stigma and severe psychiatric symptoms and poor treatment adherence [7]. Negative correlates of self-stigma include poor life satisfaction [8], impaired social relationships [9], unemployment [10,11], and poor health outcomes including poor help-seeking attitudes [12], treatment-seeking behaviors [13], and quality of life [14,15]. As self-stigma may have numerous negative effects on people with psychiatric disorders, healthcare providers and clinicians are in great need of better understanding self-stigma experienced by people in their care. Therefore, the Self-Stigma Scale-Short version (SSS-S), initially developed by Mak and Cheung to measure self-stigma in different minority groups, has been used in people with psychiatric disorders [16]. The current study aimed to further examine this instrument using Rasch modeling. By advancing the understanding of the underlying structure of the SSS-S, the psychometric properties of the SSS-S may be better established. With a better understanding of the psychometric properties of the SSS-S, results gleaned from using the instrument could be better utilized to promote the understanding and addressing of self-stigma.

Based on cognitive-behavioral theory [17], Pachankis introduced a cognitive-affective-behavioral model regarding the psychological stages of how people process stigma [18]. This process model considered three psychological domains: cognition, emotions, and behaviors. Mak and Cheung further developed the model to include concepts of self-stigma [16]. According to their framework, self-stigma may involve the three components across different groups. Specifically, when people endorse and internalize stereotypes or negative perceptions of themselves, it may result in self-stigmatizing cognitions regarding considering themselves as unworthy or less important [16]. The self-stigmatizing cognitions may generate negative effects, including feelings of inferiority, such as ignorance, anger, embarrassment, demoralization, and shame [19]. Subsequently, such negative effects may promote self-stigmatizing behaviors, such as dependency, avoidance or withdrawal from social interactions and perhaps self-sabotaging and suicidal ideation [20]. Corrigan and Rao also defined three stages of self-stigma aligning with these concepts [1]. From their perspective, individuals may become aware of the stigma and then agree that the stereotypes are true, leading to negative affective states and behaviors [21]. Göpfert et al. examined these self-stigma components in people with depression [22]. The results validated the procedural characteristics of the stages and suggested self-stigma as involving a multilevel process [23]. 

The SSS-S is a unified assessment that provides useful information regarding clients’ self-stigma. Using rigorous procedures to validate the test items within an assessment is fundamental for developing a solid measurement tool [24]. Therefore, a series of examinations were conducted to evaluate the reliability and construct validity of SSS-S in individuals with mental health concerns, immigrant women, and sexual minorities in Hong Kong [16]. Further psychometric evaluations have been conducted subsequently. For example, Wu and colleges examined the internal consistency and measurement invariance of the SSS-S across genders and across groups with different mental illnesses in Taiwan [25]. The results supported that the SSS-S was reliable and moderately and significantly associated with individuals’ depression levels and quality of life. Additionally, no measurement invariance existed within the SSS-S nine items; therefore, they concluded that clinicians could use the SSS-S with confidence across genders and different mental illnesses [25]. Another study applied both confirmatory factor analysis (CFA) and item-response theory (IRT) to simultaneously cross-validate two self-stigma assessments: the SSS-S and the Internalized Stigma Mental Illness (ISMI) scale. Both assessments were effective in measuring self-stigma in people with mental illnesses [26]. All of the above studies were conducted in Asia, a continent with high self-stigma rates [3]. The first study examining the SSS-S in the United States applied the SSS-S to 194 adults with mental illnesses from four psychosocial clubhouses [27]. The results demonstrated that SSS-S has adequate reliability and convergent and criterion validity. Additionally, the results indicated that a one-factor model fit the SSS-S items well [27]. 

Although the fundamental psychometric properties of the SSS-S have been established, Smiley suggested that clinical test theory may be used to begin assessment examinations; however, Rasch analysis is beneficial to provide further detailed information “for long-term test instrument refinement and materials development” ([28], p. 16). Rasch modeling has been extensively applied in assessment development and validation phases in healthcare research, as the Rasch techniques provide a mechanism for optimizing the test items [29]. The greatest benefit of using Rasch analysis is that it converts regular data from an ordinal scale into an interval scale of the underlying latent trait. In this case, the latent construct of the self-stigma within the SSS-S can be compared linearly when the person’s ability/tendencies and the item’s difficulty are aligned [24]. Therefore, the current study aimed to further examine the psychometric properties of the SSS-S with Rasch analysis. Specifically, Rasch analysis was used to examine the SSS-S’ 4-point rating scale functioning, item hierarchy, unidimensionality, and person-response validity. Additionally, as self-stigma may not be equally salient across different stigmatized groups, the differential item functioning (DIF) of the SSS-S across different groups needs to be examined empirically [16]. That said, DIF in the Rasch analysis may help researchers and healthcare providers understand whether people with different psychiatric diagnoses (e.g., substance use disorders (SUDs), attention-deficit/hyperactivity disorder (ADHD), and schizophrenia (SZ), as in the current study) may similarly or differentially experience aspects of self-stigma. Lastly, the concurrent validity of the SSS-S has only been examined in limited fashions with other assessments: the Perceived Stigma toward People who use Substances (PSPS) [30], Depression and Somatic Symptoms Scale (DSSS), and WHOQOL-BREF [25]. Here, the SSS-S’ concurrent validity with the Depression, Anxiety, Stress Scale (DASS-21) was examined as the DASS-21 has been translated into 54 languages, which allows it to be widely used globally [31].

With Rasch analysis, a more comprehensive understanding of the SSS-S items was anticipated, and this understanding could include results of a detailed comparison of participants’ ability/tendencies and items’ difficulty on a linear continuum. Furthermore, the structure of the SSS-S rating scale and DIF across three different diagnoses would be produced, providing potentially complementary information derived from other approaches [32].

## 2. Materials and Methods 

### 2.1. Study Site

All participants in the current study were recruited from one psychiatric center, the Jianan Psychiatric Center (JPC). The JPC is the largest psychiatric center in Taiwan, and it provides mental health services and psychiatric treatment to people residing in southern Taiwan. Therefore, the JPC could refer to the current study a sufficient number of participants with psychiatric diagnoses of SUDs, ADHD, and SZ [30,33,34].

The recruitment of the current study’s participants followed rigorous procedures: (i) the JPC psychiatrists evaluated the participants’ eligibility regarding whether they fulfilled the inclusion and exclusion criteria mentioned in the Participants section below; (ii) the psychiatrists referred the potential participants to the research assistants to deliver the information regarding the study purpose and participants rights; (iii) the research assistants asked the participants who were interested in the current study to sign a written informed consent (for those who were aged under 21 years, their legal guardian also signed a written informed consent after understanding the study); (iv) the research assistants led the participants to a quiet room without disturbance and gave them the measurements (i.e., the SSS-S and DASS-21) to complete; (v) the research assistants supervised and answered questions while participants were completing the measurements; (vi) the research assistants collected the measurements and checked the completeness of the measurements; (vii) the research assistants asked the participants to complete any missing items, if any. 

Given that this study involved individuals with psychiatric disorders, ethics should be carefully considered, and the associated guidelines should be strictly followed to protect the rights of participants as well as safeguard the interests of the research team [35]. Considering individuals with psychiatric disorders as potentially vulnerable, the informed consent was carefully developed with layman terms so it could be easily understood by the study participants. Additionally, the research assistants made extra efforts to answer any questions and ensure the participants understood the information provided and made autonomous decisions. 

This study was not preregistered, and the JPC institutional review board approved the current study before study commencement with the following registered numbers: Ref no. 18-039, 19-034, and 20-026. 

### 2.2. Participants 

**Participants with substance use disorders (SUDs).** People with SUDs (including opioid, amphetamine or alcohol use disorders) who were diagnosed according to the DSM-5 diagnostic criteria were recruited from the addiction outpatient clinics of the JPC [36]. Apart from diagnoses of SUDs, eligible participants also met the following inclusion criteria: (i) were over 20 years old, and (ii) understood the measurements used in the current study. SUD participants with the following diagnoses were excluded: (i) intellectual disabilities; (ii) dementia; and (iii) SZ or other psychosis.

**Participants with attention deficit hyperactivity disorder (ADHD).** People with ADHD who were diagnosed according to the DSM-5 diagnostic criteria were recruited from child and adolescent psychiatry outpatient clinics of the JPC [36]. Apart from the diagnosis of ADHD, eligible participants also met the following inclusion criteria: (i) aged between 7 and 20 years, inclusive, and (ii) understood the measurements used in the current study. ADHD participants with the following diagnoses were excluded: (i) intellectual disabilities; (ii) epilepsy, (iii) major psychotic disorder; and (iv) autism spectrum disorder.

**Participants with schizophrenia (SZ).** People with SZ who were diagnosed according to the DSM-5 diagnostic criteria were recruited from partial hospitalization (daycare) and general psychiatry outpatient clinics of the JPC [36]. Apart from the diagnosis of SZ, eligible participants also met the following inclusion criteria: (i) were over 20 years old, and (ii) understood the measurements used in the current study. SZ participants with the following diagnoses were excluded: (i) SUDs; (ii) dementia; and (iii) head injury.

### 2.3. Measures

**Self-Stigma Scale-Short (SSS-S).** The Self-Stigma Scale (SSS-S) is a widely used assessment to evaluate internalized stigma. The SSS-S consists of nine items in three subdomains (i.e., self-stigma in cognition, affect, and behavior). Each item is self-reported on a 4-point Likert scale (1 = very much disagree, 2 = disagree, 3 = agree, and 4 = very much agree), with higher scores representing higher levels of self-stigma. Given that the assessment was designed to be used with different minority populations, the terminology describing the minority group in the SSS-S may be replaced based on the study population [16]. Specifically, people with SUDs, people with ADHD, and people with SZ participated in the current study. All nine SSS-S items can be found in Table 1.

**Depression, Anxiety, Stress Scale (DASS-21)**. The Depression, Anxiety and Stress Scale (DASS) is a commonly used questionnaire for evaluating individuals’ self-reported depression, anxiety, and stress [37]. The current study used the DASS-21. Each item in the DASS-21 is scored on a 4-point Likert scale ranging from 0 (“did not apply to me at all”) to 3 (“applied to me very much”), with higher scores reflecting higher levels of depression, anxiety, or stress. The DASS-21 items can be found in Table 1.

### 2.4. Data Analysis

Rasch analyses were used to examine the SSS-S’ scale functioning, unidimensionality, person-response validity and DIF across the three subgroups (i.e., people with SUDs, ADHD, or SZ). Given that previous studies had supported a one-factor model for the SSS-S [16,26,27], all three subdomains of the nine items were combined into one Rasch analysis. Facets Version 3.83.6 and Winsteps Version 5.2.2 were used to perform the Rasch analysis. Additionally, a one-way ANOVA was used to compare self-stigma, depression, anxiety, and stress across the three diagnoses. Post hoc pairwise comparison was further conducted to examine differences between subgroups with Bonferroni correction of *p* < 0.017 (0.05/3). SPSS version 27 was used for generating descriptive statistics and investigating concurrent validity. 

**Rating Scale Functioning and Item Hierarchy.** The 4-point Likert scale of the SSS-S was investigated first. Sufficient subject enrollment was required to achieve stable outcomes in the Rasch analysis; therefore, with all nine items, it was expected that there would be more than 10 participants in each rating category. Additionally, whether the average calibrations increased monotonically across the 4-point Likert scale in the SSS-S was investigated. Further, the outfit mean squared (MnSq), which was sensitive to unexpected outliers, was monitored for the SSS-S. The criterion was that the Outfit MnSq should remain less than 2 for all nine items. Finally, the item hierarchy of the SSS-S was examined.

**Unidimensionality.** The unidimensionality of the SSS-S was examined using the goodness-of-fit statistics results from the Rasch analysis for the combined nine items. Rasch fit statistics represent the fit of SSS-S items to the Rasch model. The mean square fit statistics were expected to be the value of 1, while the fit statistics over or less than 1 would be interpreted as overfit (i.e., more variation/noise than predicted by the Rasch model) or underfit (i.e., less variation/overlap than predicted by the Rasch model), respectively. The infit MnSq was expected in the range of 0.6 to 1.4 with a standardized mean square (Zstd) between −2 to +2. According to the Rasch measurement model, if the infit MnSq values are within the range, suggesting minimal distortion of the scoring, then the Zstd may be ignored. However, one should be cautious if an item’s infit MnSq value is over 1.4, suggesting that the item potentially deviates from unidimensionality, and thus it should be further investigated. Additionally, a principal components analysis (PCA) of the residuals was conducted to further examine the underlying structure. An eigenvalue of the first contrast was expected to be less than 3.

**Person-Response Validity.** Next, the authors used Rasch analysis to examine how well the enrolled participants fit into the Rasch model’s expectation. The criteria were similar to the unidimensionality examination, in that the infit and outfit MnSq of the enrolled participants should range between 0.4 and 1.6 with a Zstd between −2 and +2. Moreover, 95% of the enrolled participants should have acceptable goodness-of-fit to confirm the person-response validity. Additionally, extreme values of the responses from the SSS-S were examined for ceiling and floor effects. The criteria were set so that if more than 15% of the total enrolled participants received the maximum or minimum possible scores on the SSS-S, then they were considered as ceiling or floor effects, respectively.

**Differential Item Functioning (DIF).** As the current study investigated the SSS-S across three diagnostic groups (i.e., SUDs, ADHD, and SZ), DIF analysis was employed to examine whether specific items performed consistently across different subgroups. Specifically in this study, DIF examined whether participants with different diagnoses who have a similar underlying latent feature of self-stigma may have different probabilities to endorse any of the SSS-S items. If less than 5% of items in a longer questionnaire or no more than one item in a shorter questionnaire exhibited DIF, then it suggests that there was no additional construct that had an influence on the intended construct of the measurement, self-stigma. As the SSS-S has only nine items, it was expected that there should be no DIF item across the three diagnoses. The DIF contrasts results from the Rasch analysis were used as the evaluation criteria. A DIF contrast over 1 suggests significant DIF.

**Concurrent Validity.** Concurrent validity of the SSS-S was examined with the DASS-21. Due to the nature of the ordinal scales for both the SSS-S and DASS-21, the Spearman correlation was conducted with the criteria such that 0.1 to 0.3 = weak correlation; 0.4 to 0.7 = moderate correlation; > 0.7 = strong correlation.

## 3. Results

### 3.1. Participant Demographics

**Figure 1:**
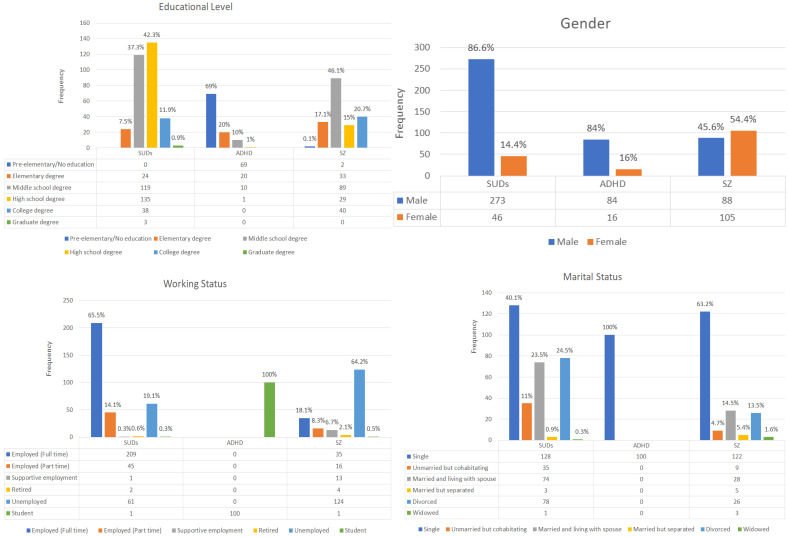
Participants’ characteristics (*N* = 612).

The study had 612 participants; among them, 319 were diagnosed with SUDs, 100 with ADHD, and 193 with SZ. The mean ages were similar between participants with SUDs (mean = 42.2 years; S.D. = 8.9) and those with SZ (mean age = 41.3 years, S.D. = 9), and the participants with ADHD were mainly children (mean age = 10.8 years, S.D. = 3.1). The majority of participants with SUDs (87%) and those with ADHD (84%) were male. Other demographic information can be found in Figure 1. Item scores for the SSS-S and DASS across the diagnostic groups can be found in Table 1. In general, ANOVA results showed significant differences across groups in both the SSS-S and DASS-21. Post hoc pairwise comparisons suggested that participants with SUDs (*p* < 0.001) and those with SZ (*p* < 0.001) had higher levels of self-stigma compared to those with ADHD. Additionally, participants with SZ had the highest levels of depression and anxiety, and these were different from participants with SUDs (*p* < 0.001) and ADHD (*p* < 0.001). Moreover, the SZ population had higher stress than the SUD population (*p* < 0.001); no differences in stress condition were found between participants with SZ and ADHD (*p* = 0.572) or SUDs and ADHD (*p* = 0.140).

### 3.2. Rating Scale Functioning and Item Hierarchy

**Figure 2:**
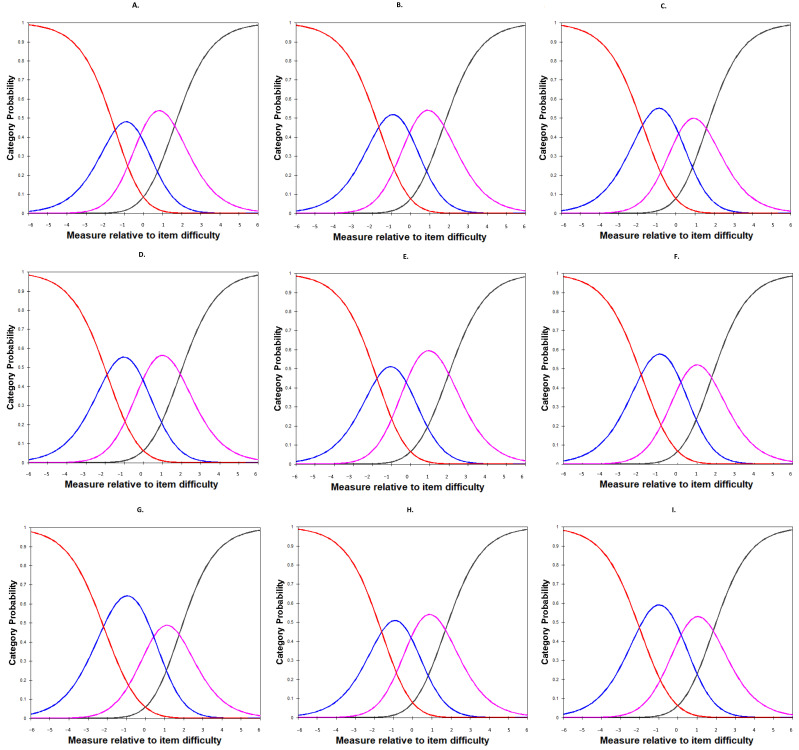
Probability curves of the nine Self-Stigma Scale-Short (SSS-S) items. Note. Red lines indicate the probabilities of answering with the response “very much disagree (1)”; blue lines indicate the response “disagree (2)”; purple lines indicate the response “agree (3)”; black lines indicate the response “very much agree (3)”. In Figure 2, each SSS-S item showed clearly how the rating thresholds were properly ordered. Each rating category (1, 2, 3 and 4) has a peak in the curve along the expected self-stigma continuum. The subfigures (**A**–**I**) are corresponding to the 9 items in SSS-S. Details of each item can be found in Table 1.

All nine items of the SSS-S had over 10 subjects in all of the 4-point rating scales, with the minimum endorsement being the highest severity option (i.e., 4 = very much agree) in seven of the nine questions. All outfit MnSq values for the nine items of the SSS-S were less than 2, and the calibration of each rating category advanced monotonically. Overall, items in the cognitive subdomain were more robustly endorsed with lower Rasch calibrations (−0.82 to −0.29), and the items in the behavioral subdomain were less robustly endorsed with higher Rasch calibrations (0.32 to 0.69). Among the nine items, the most robustly endorsed item was item 2 (*My identity as a ______incurs inconvenience in my daily life.*) from the cognitive self-stigma domain. The most robustly endorsed item was item 7 (*I feel like I cannot do anything about my _____ status.*) from the affective self-stigma domain. Probability curves of the SSS-S items can be found in Figure 2.

### 3.3. Unidimensionality

**Figure 3:**
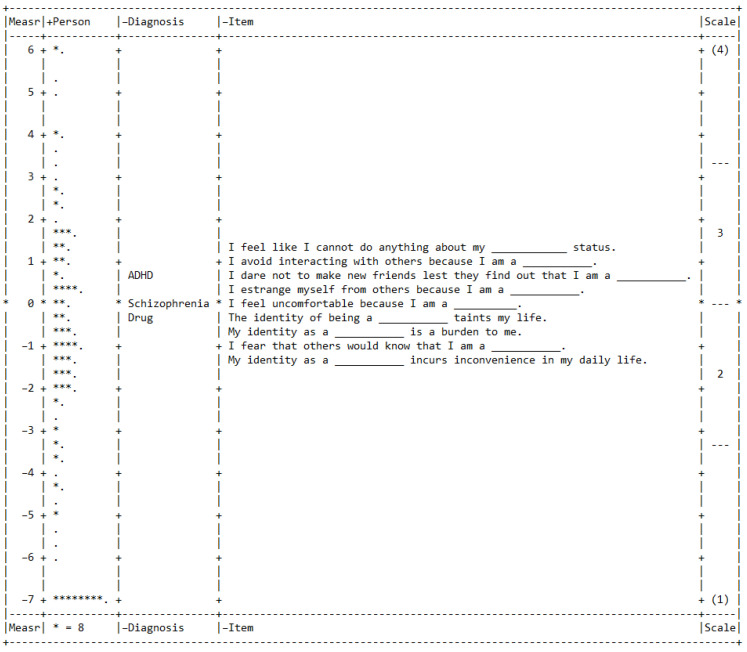
Wright map of the person, diagnosis and the SSS-S items. Each “ * ” represents 8 persons; each “ . ” represents 1 to 7 persons.

**Figure 4:**
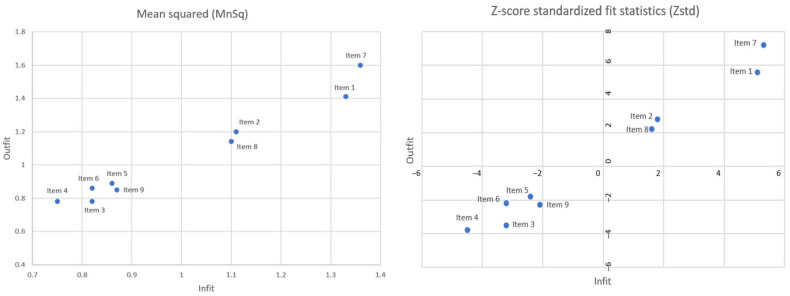
Rasch mean square (MnSq) and Z-score standardized fit statistics (Zstd) of the Self-Stigma Scale-Short (SSS-S).

All nine items had Infit MnSq values ranging from 0.4 to 1.6. Additionally, the Rasch dimension explained 54.4% of the total variance in the data. The PCA of the residual results showed that the first unexplained variance had an eigenvalue of 2.1. Figure 3 shows a Wright map, and detailed Infit MnSq and Zstd statistics can be found in Figure 4.

### 3.4. Person-Response Validity

There were 85 enrolled participants (13.9%) whose data misfit the Rasch model. Among them, 14.5%, 16%, and 12.5% of participants misfit from the SUD, ADHD, and SZ populations, respectively. In addition, 11 (1.8%) and 71 (11.6%) participants self-reported the maximum and minimum scores on the SSS-S. 

### 3.5. Differential Item Functioning

Two SSS-S items demonstrated DIF contrasts over 1. Item 3 (*I dare not to make new friends lest they find out that I am a ____.*) from the Behavior domain had a DIF contrast of −1.14 (SUDs vs. ADHD) and 1.41 (ADHD vs. SZ). Rasch calibration = 0.05 for SUDs, 0.63 for SZ, and 1.64 for ADHD. Item 7 (*I feel like I cannot do anything about my ____ status.*) from the Affect domain also demonstrated DIF contrasts of 1.77 (SUDs vs. ADHD) and −1.05 (ADHD vs. SZ). Rasch calibration = 1.81 for SUDs, 1.09 for SZ, and 0.04 for ADHD.

### 3.6. Concurrent Validity

**Figure 5:**
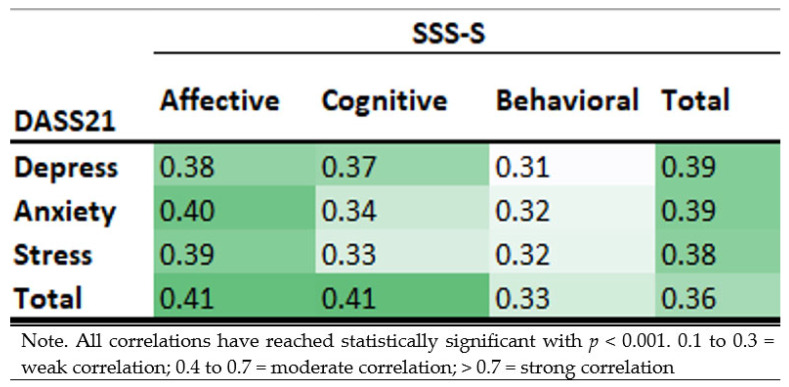
Spearman’s correlations between Self-Stigma Scale-Short (SSS-S) and Depression, Anxiety and Stress (DASS-21) scores.

The SSS-S subdomains and the total scores had weak to marginally moderate correlation with the depression, anxiety and stress subdomains and the total scores (r = 0.31 to 0.41, *p* < 0.001). Further details can be found in Figure 5.

## 4. Discussion

The current study examined the psychometric properties of a commonly used self-reported self-stigma questionnaire, the Self-Stigma Scale-Short version (SSS-S), among three different psychiatric populations in Taiwan. A prior study evaluated the psychometric properties and utilization of the SSS-S in people with mental health issues, immigrants, and sexual minorities [16]. The current study provides empirical evidence of the construct validity of the SSS-S among individuals with SUDs, ADHD, and SZ. Additionally, to the best of the authors’ knowledge, this is the first study to examine the SSS-S with Rasch analysis across groups with different psychiatric diagnoses. Rasch results demonstrated satisfactory goodness-of-fit, which confirmed the unidimensionality of the SSS-S. The four-point Likert scale of the SSS-S reflected monotonical increases along the self-stigma continuum. Cognitive items within the SSS-S were the most strongly endorsed, while the behavioral items were the least strongly endorsed. Less than 15% of participants misfit the Rasch model; therefore, no ceiling and floor effects were detected in the SSS-S. Two items displayed DIF across diagnoses. Lastly, SSS-S scores correlated weakly to moderately with DASS-21 scores.

The Rasch results confirmed that all nine items in the SSS-S advanced monotonically along a self-stigma continuum. All nine items had proper Infit MnSq values, which confirmed the unidimensionality. This Rasch result was also consistent with prior studies suggesting a single factor [16,26,27]. Prior studies have also confirmed a second-order structure of the SSS-S construct [16,25], with cognitive, affective and behavioral components representing a generalized self-devaluation for people with high levels of self-stigma. The current study advanced the concepts and provided item calibrations from Rasch analysis to further demonstrate the self-stigma hierarchy. Specifically, across the three psychiatric diagnoses included in the current study, cognitive items were most robustly endorsed, and behavioral items were least robustly endorsed. The results suggest that it may be beneficial for healthcare clinicians to detect potential self-stigma at early stages if they notice evidence of cognitive aspects of self-stigma being endorsed by patients, and this possibility warrants further examination.

In the current study, two SSS-S items displayed DIF. One was from the Behavior subdomain (i.e., item 3) and the other from the Affect subdomain (i.e., item 7). Compared to participants with ADHD, participants with SUDs and SZ more strongly endorsed item 3 (i.e., *I dare not to make new friends lest they find out that I am a ____.*), suggesting that they had higher levels of self-stigma that may have prevented them from making new friends. Another item that displayed DIF was item 7 (*I feel like I cannot do anything about my ____ status.*). Specifically, compared to participants with SUDs and SZ, participants with ADHD more strongly endorsed this item, suggesting that they may have felt more apathy or lower levels of control over their ADHD status. Additionally, developmental influences regarding perceived control over situations should be considered when interpreting the findings. 

Regarding the DIF of item 3, this result was consistent with a recent study finding that many individuals with psychiatric disorders lost social contacts and perceived friendships changed as their illnesses developed or progressed [38]. Specifically, one study showed that 71% of enrolled participants indicated that social contacts had been reduced because of their self-stigma; that is, the participants anticipated a negative reaction from others that resulted in social withdrawal [38]. A systematic review revealed that individuals with mental illness had an average of 3.4 individuals in their friendship networks [39]. Another study specifically explored the quantitative aspect of friendship in people with SZ and found the mean number of friends reported was 1.57 [40]. Regarding the DIF of item 7, the result was consistent with previous studies showing that individuals with ADHD feel of unpredictability and lack of control daily [41]. Common stereotypes about people with ADHD as lazy and lacking motivation may also contribute to their self-stigma [42,43]. 

The current study showed that participants with SUDs and those with SZ had significantly higher self-stigma compared to those with ADHD. Additionally, the SZ population had the highest levels of depression, anxiety, and stress. The results add to findings in the literature that people with SZ may be particularly vulnerable to self-stigma. Chang et al. found that people with SZ and those with bipolar disorder had significantly higher levels of self-stigma compared to people with anxiety disorders [44]. In addition, Wu et al. found that people with SZ tended to have higher self-stigma compared to people with other mental illnesses [25]. This finding also aligned with previous studies that concluded people with increased severity of symptoms experienced higher levels of self-stigma [7,45]. Therefore, results across studies suggest that people with SZ may be particularly likely to internalize stigma and develop high levels of self-stigma. 

According to Corrigan and Rao, people in a stigmatized group do not necessarily internalize public stigma [1]. They can be aware of social prejudice but not cognitively believe it, nor would it necessarily influence their emotions or behaviors. Therefore, the SSS-S is valuable as a reliable and valid assessment for healthcare clinicians for early detection of potential self-stigma. With such information, appropriate advocacy may be enacted, and interventions developed and implemented. For example, Drapalski et al. conducted a randomized controlled trial with a group intervention that sought to decrease self-stigma in veterans with mental illness [46]. They found reduction of self-stigma from baseline to post-intervention, and the reduction was maintained at the 6-month follow-up [46].

Apart from the SSS-S, other existing self-stigma measures could be considered for use. Specifically, the Internalized Stigma of Mental Illness (ISMI) [47,48] and Self-Stigma of Mental Illness Scale (SSMIS) [49] are both validated instruments assessing self-stigma in people with psychiatric concerns. 

### Limitations and Future Study Suggestions

This study had several limitations, so the results should be interpreted with caution. First, the severity of symptoms may not have been comparable between the three psychiatric illnesses and could not be controlled because no universal index was assessed for all participants regarding their symptom severity in the current study. Higher levels of negative symptoms and hostility have been associated with poor friendships in people with mental illness [50]. Thus, symptom severity could have been a confounder in the current study. Second, the participants with ADHD were younger than those with SUDs and SZ. Therefore, participants’ daily occupations, life experiences, social encounters and brain development linked to processing stigma may have differed because of their corresponding life stages. Future studies should examine how the different life stages may impact perceived self-stigma. Last, self-stigma can be dynamic given changes in life events and encounters. Longitudinal studies should be conducted to better understand the patterns/cycles of self-stigma in people with psychiatric disorders. Further investigations could also consider using regression or structural equation models to investigate across different diagnostic groups potential life events or other factors that may influence self-stigma. 

## 5. Conclusions

The SSS-S is a short, sound assessment tool designed to evaluate self-stigma across different minority groups. The current study applied Rasch analyses and confirmed the SSS-S’ unidimensionality when evaluating self-stigma in people with SUDs, ADHD, and SZ. All nine SSS-S items have appropriate rating scale functioning, which properly captured certain aspects of self-stigma. Furthermore, the different diagnostic populations all appeared to have experienced self-stigma, although different severities were observed. Given that self-stigma remains a serious issue for individuals with mental illness and may lead to adverse consequences, validating the SSS-S is an important step towards accurate evaluation. Given the promising results from the Rasch analyses, clinicians should use the SSS-S regularly and early to detect potential self-stigma and utilize interventions to reduce self-stigma in individuals in psychiatric care. 

## Figures and Tables

**Table 1:** Item Descriptions and Scores Across The Three Diagnostic Groups (*N* = 612).

Variables	M (S. D.)			F
	SUDs (*n* = 319)	ADHD (*n* = 100)	SZ (*n* = 193)	
**SSS-S Items**				
I fear that others would know that I am a ___________.	2.73 (1.07)	1.85 (1.02)	2.64 (1.02)	27.5 **
My identity as a ___________ incurs inconvenience in my daily life.	2.85 (1.03)	1.98 (1.08)	2.49 (1.02)	28.2 **
I dare not to make new friends lest they find out that I am a ___________.	2.23 (1.02)	1.35 (0.67)	2.26 (0.96)	36.8 **
I feel uncomfortable because I am a __________.	2.35 (1.02)	1.68 (0.85)	2.41 (0.96)	21.3 **
My identity as a ___________ is a burden to me.	2.67 (1.03)	1.79 (0.92)	2.55 (0.96)	30.4 **
I estrange myself from others because I am a ___________.	2.27 (1.00)	1.59 (0.82)	2.26 (0.97)	20.7 **
I feel like I cannot do anything about my ____________ status.	1.97 (0.90)	1.77 (0.95)	2.06 (0.88)	3.4 **
The identity of being a ___________ taints my life.	2.63 (1.05)	1.69 (0.93)	2.34 (0.98)	33.4 **
I avoid interacting with others because I am a ___________.	2.16 (0.95)	1.69 (0.73)	2.10 (0.92)	27.4 **
**DASS Items**				
*DASS-Depress Domain Total Scores*	8.05 (10.50)	7.98 (7.42)	11.80 (10.92)	9.02 **
I couldn’t seem to experience any positive feeling at all	0.64 (0.92)	0.59 (0.78)	0.93 (1.03)	6.9 **
I found it difficult to work up the initiative to do things	0.47 (0.84)	1.28 (1.06)	0.92 (1.06)	32.7 **
I felt that I had nothing to look forward to	0.72 (0.99)	0.64 (0.95)	1.03 (1.07)	7.4 **
I felt down-hearted and blue	0.66 (0.95)	0.53 (0.80)	1.00 (1.05)	10.6 **
I was unable to become enthusiastic about anything	0.54 (0.86)	0.43 (0.87)	0.78 (0.96)	6.2 *
I felt I wasn’t worth much as a person	0.47 (0.89)	0.17 (0.57)	0.56 (0.92)	7.2 **
I felt that life was meaningless	0.54 (0.91)	0.35 (0.70)	0.68 (1.02)	4.4 *
*DASS-Anxiety Domain Total Scores*	7.05 (8.64)	6.32 (7.26)	11.44 (10.52)	17.0 **
I was aware of dryness of my mouth	0.80 (0.92)	0.63 (0.88)	1.31 (1.13)	22.2 *
I experienced breathing difficulty	0.39 (0.76)	0.29 (0.64)	0.62 (0.97)	6.7 **
I experienced trembling	0.47 (0.83)	0.35 (0.80)	0.53 (0.82)	1.66
I was worried about situations in which I might panic and make a fool of myself	0.67 (0.93)	0.83 (1.06)	1.03 (1.08)	7.7 **
I felt I was close to panic	0.40 (0.78)	0.49 (0.86)	0.73 (0.96)	8.8 **
I was aware of the action of my heart in the absence of physical exertion	0.42 (0.76)	0.23 (0.66)	0.81 (1.05)	19.3 **
I felt scared without any good reason	0.39 (0.76)	0.34 (0.79)	0.70 (1.00)	9.6 **
*DASS-Stress Domain Total Scores*	9.40 (10.92)	11.90 (8.34)	13.67 (12.14)	9.4 **
I found it hard to wind down	0.61 (0.87)	1.17 (1.03)	0.95 (1.09)	15.7 **
I tended to over-react to situations	0.53 (0.83)	0.70 (0.89)	1.04 (1.00)	16.8 **
I felt that I was using a lot of nervous energy	0.74 (0.94)	0.63 (0.85)	1.15 (1.06)	13.7 **
I found myself getting agitated	0.64 (0.93)	0.51 (0.81)	0.88 (1.05)	6.2 *
I found it difficult to relax	0.72 (0.97)	0.65 (0.93)	0.99 (1.11)	5.7 *
I was intolerant of anything that kept me from getting on with what I was doing	0.78 (2.01)	1.08 (1.06)	0.88 (1.02)	1.3
I felt that I was rather touchy	0.70 (0.91)	1.21 (1.12)	0.98 (2.53)	4.4 *

Note 1. SUDs = Substance Use Disorders; ADHD = Attention-Deficit/Hyperactivity Disorder; SZ = Schizophrenia; M = Mean; S.D. = Standard Deviation. ** *p* < 0.001; * *p* < 0.05. Note 2. ___________ in the SSS-S items was replaced by different terminology (i.e., SUDs, ADHD, and SZ) that was applicable in each population.

## Data Availability

The datasets generated and/or analyzed during the current study are available from the corresponding author on reasonable request.
